# The Conformational Changes of Bovine Serum Albumin at the Air/Water Interface: HDX-MS and Interfacial Rheology Analysis

**DOI:** 10.3390/foods12081601

**Published:** 2023-04-10

**Authors:** Fei Han, Qian Shen, Wei Zheng, Jingnan Zuo, Xinyu Zhu, Jingwen Li, Chao Peng, Bin Li, Yijie Chen

**Affiliations:** 1 College of Food Science and Technology, Huazhong Agricultural University, Wuhan 430070, China; 2 National Facility for Protein Science in Shanghai, Shanghai Advanced Research Institute, Chinese Academy of Sciences, Shanghai 201210, China; 3 Shenzhen Institute of Nutrition and Health, Huazhong Agricultural University, Wuhan 430070, China; 4 Genome Analysis Laboratory of the Ministry of Agriculture, Agricultural Genomics Institute at Shenzhen, Chinese Academy of Agricultural Sciences, Shenzhen 518124, China

**Keywords:** protein, air/water interface, conformational changes, hydrogen–deuterium exchange mass spectrometry, interfacial rheology

## Abstract

The characterization and dynamics of protein structures upon adsorption at the air/water interface are important for understanding the mechanism of the foamability of proteins. Hydrogen–deuterium exchange, coupled with mass spectrometry (HDX-MS), is an advantageous technique for providing conformational information for proteins. In this work, an air/water interface, HDX-MS, for the adsorbed proteins at the interface was developed. The model protein bovine serum albumin (BSA) was deuterium-labeled at the air/water interface in situ for different predetermined times (10 min and 4 h), and then the resulting mass shifts were analyzed by MS. The results indicated that peptides 54–63, 227–236, and 355–366 of BSA might be involved in the adsorption to the air/water interface. Moreover, the residues L55, H63, R232, A233, L234, K235, A236, R359, and V366 of these peptides might interact with the air/water interface through hydrophobic and electrostatic interactions. Meanwhile, the results showed that conformational changes of peptides 54–63, 227–236, and 355–366 could lead to structural changes in their surrounding peptides, 204–208 and 349–354, which could cause the reduction of the content of helical structures in the rearrangement process of interfacial proteins. Therefore, our air/water interface HDX-MS method could provide new and meaningful insights into the spatial conformational changes of proteins at the air/water interface, which could help us to further understand the mechanism of protein foaming properties.

## 1. Introduction

Proteins are amphiphilic biopolymers that can quickly adsorb to the air/water interface, reduce surface tension, and further stabilize foam-based food systems [1]. The solubility, hydrophobicity, flexibility, and charge of protein molecules are the major factors impacting the interfacial adsorption process [2,3,4]. The interfacial adsorption of proteins includes two basic steps: (i) the protein molecules move rapidly to the air/water interface and their attachment; (ii) the polymer chains uncoil and rearrange (surface denaturation) [5]. Previous research has demonstrated that proteins adsorb to the air/water interface by hydrophobic and electrostatic interactions [6]. Therefore, hydrophobic and positively charged amino acid residues are the potential sites interacting with the interface. After the adsorption of proteins at the interface, changes are achieved not only quantitatively but also conformationally [7,8].

The degree of conformational changes of proteins at the air/water interface strongly affects the rheological properties of the interfacial films formed by proteins [9,10]. The degree of conformational changes of globulins is low, and the viscoelastic modulus of interfacial films is high. On the contrary, flexible proteins exhibit more conformational changes at the air/water interface, resulting in a lower viscoelastic modulus of the interfacial films [11,12,13]. The rheological properties of interfacial films further influence the properties of foams stabilized by proteins [6]. The higher viscoelastic modulus of interfacial films contributes to higher foam stability [14,15]. Thus, it is of great significance to study the conformational changes of proteins at the air/water interface, which is beneficial to further understand the properties of macroscopic foams and develop foam-based food systems. The conformational changes of flexible and structured proteins at the air/water interface are different. The typical flexible protein, β-casein, presented a conformation of a random coil in solution and transformed to a β-sheet structure after adsorbing to the air/water interface via in situ circular dichroism (CD) spectroscopy [16] and infrared reflection–absorption spectroscopy (IRRAS) [17]. However, changes in the secondary structure of structured proteins were often accompanied by an increase in random coils or β-sheets, and a decrease in α-helices [18]. For example, bovine serum albumin (BSA), the typical structured globular protein, missed its helical structure and transformed into a random coil at the air/water interface studied by in situ CD spectroscopy [16]. Lysozyme, another structured protein, was an α+β-type protein in the solution. It transformed its α-helix into a β-sheet upon adsorption to the air/water interface, as revealed by external reflection Fourier-transform infrared (ER-FTIR) spectroscopy [19]. However, these spectral results can only reflect the conformational transitions of proteins adsorbed to the interface at the secondary structure level. The information on the influence of the interface on the protein structure at the peptide level and their interaction sites is still unclear. Thus, an accurate and effective method to determine the structural changes of proteins at the air/water interface in situ at the peptide and amino acid residue level is urgently needed. Hydrogen–deuterium exchange coupled with mass spectrometry (HDX-MS) is a promising technique to monitor conformational changes of proteins. It is capable of overcoming the disadvantage of the techniques above. Its principle is that amide hydrogens of the protein backbone exchange with deuterium atoms in the surrounding solvent over defined periods of time. Since the mass of deuterium is roughly double the mass of hydrogen, the resulting mass shifts can be determined by MS to monitor the rate of HDX, which can provide information on the solvent accessibility of the protein, and further information on the protein structure [20,21,22]. Hydrogens on the surface of the protein that are more exposed to D_2_O demonstrate a faster HDX rate than those located inside or involved in hydrogen bonding [23,24]. As the conformation of proteins influences the rate of HDX in proteins, monitoring the deuteration level of proteins over time can reveal the characteristics of protein conformation and conformational changes under the influence of a variety of factors: protein interactions, ligands, surface denaturation, etc. [25]. Compared to the spectral methods, CD and FTIR, which can only reflect structural changes of proteins at the secondary structure level, HDX-MS can provide a resolution of the conformational changes of proteins at the peptide and amino acid residue level.

Proteins can interact with proteins, ligands, and membranes, resulting in a decreased HDX rate on interaction sites. Thus, we can monitor the HDX rate to identify the interaction sites. The HDX-MS analysis of IL-23R with its cognate cytokine IL-23 indicated that, compared to the HDX rate without IL-23, two peptides (residues 24–42 and 88–113) located in the N-terminal showed a lower HDX rate. Therefore, residues 24–42 and 88–113 of IL-23R might be the main sites between IL-23R and IL-23 interaction [26]. After the binding of AMP to AMP-activated protein kinase (AMPK), the conformational changes of AMPK mainly happened to the γ-subunit [27]. In order to analyze conformational information for membrane-associated HIV-1 Nef protein, a novel type of HDX-MS utilization was developed, combined with Langmuir monolayers [28]. However, the HDX-MS study of BSA was limited. The researchers used HDX-MS to study conformational changes of ultrasonicated BSA, and the structural unfolding of BSA after ultrasonication was revealed [29]. Here, we applied HDX-MS for food research to explore conformational changes and interaction sites of BSA upon adsorption to the air/water interface.

In this article, BSA was used as the model protein to develop an HDX-MS method to monitor the conformational transitions of proteins at the air/water interface in situ. Furthermore, we combined information about protein structural changes with its interfacial adsorption properties to clarify the molecular mechanism of protein foaming properties, establishing the relationship between the structure and foaming function of proteins. We hope our work provides theoretical support for the development of foam-based food products that are stabilized by proteins.

## 2. Materials and Methods

### 2.1. Materials

Bovine serum albumin (≥98%, Catalog No. V900933), pepsin (porcine gastric mucosa, Catalog No. P6887), aspergillopepsin (Protease from Aspergillus saitoi, Catalog No. P2143), and guanidine hydrochloride (≥99%, Catalog No. G3272) were purchased from Sigma-Aldrich (St. Louis, MO, USA). Deuterium oxide (99.9%, Catalog No. R002863) was obtained from RHAWN (Shanghai, China). Formic acid was provided by Fluka (Seelze, Germany). All the reagents were dispersed in ultrapure water (Milli-Q, Millipore Corporation, Bedford, MA, USA).

### 2.2. Solution Preparation

The BSA solution was prepared in phosphate buffer saline (PBS, 10 mM, pH 7) and stirred for 2 h at room temperature. The BSA dispersion was maintained at 4 °C for 12 h for full hydration.

### 2.3. Foaming Properties

The optimal protein concentration for the interfacial experiments was determined according to the foaming properties. With a homogenizer (T18, IKA) set to 8000 rpm, BSA solutions under the various concentrations (1, 3, 5, 7, and 9%, *m*/*v*, 20 mL) were foamed for 2 min at room temperature. The foams were poured into 50 mL measuring cylinders as soon as possible. According to an earlier method, foamability (FA) and foam stability (FS) calculations were made [30]. In short, FA was calculated by comparing the foam volume at 2 min to the beginning liquid volume of the samples (20 mL), whereas FS was evaluated by comparing the foam volume at 60 min to the foam volume at 2 min.

(1) $$FA=\frac{V_{2}}{20}\times 100\%$$

(2) $$FS=\frac{V_{60}}{V_{2}}\times 100\%$$

where V_2_ is the volume of foam at 2 min, and V_60_ is the volume of foam at 60 min.

On a Nikon instrument, the visual foam volume was captured in photos after being kept at room temperature for 2, 5, 10, 15, 30, 45, 60, and 90 min. Three measurements were made for FA and FS.

### 2.4. Interfacial Rheology Analysis

The interfacial rheology of the BSA at the air/water interface was determined by a drop tensiometer (Tracker Teclis/IT Concept, Longessaigne, France) [31]. Following the injection of the BSA solution into a vessel, an axisymmetric rising bubble with a constant volume of 4 μL was produced at the inverted tip of an air-filled syringe. A camera with a charge-coupled device (CCD) was adopted to capture the photographs of the bubble. It took 7200 s to measure each sample. The surface tension of the PBS was measured to make sure there were no additional surfactants present. The interfacial pressure (π) was obtained from Equation (3)

(3) $$\pi =\gamma^{0}-\gamma^{s}$$

where π represents the interfacial pressure, γ^0^ represents the interfacial tension of the PBS, and γ^s^ represents the interfacial tension of the protein solution. At a constant temperature of 25 °C ± 1 °C, at least three replicates of the measurements were made for each sample.

### 2.5. Hydrogen–Deuterium Exchange in the Solution

Purchased deuterium oxide (RHAWN, Shanghai, China) was used directly to carry out the HDX experiments. To initiate the HDX experiments in the solution, 10 μL of the prepared BSA solution (0.1%, *m*/*v*) was added to 140 μL of D_2_O and allowed to be labeled continuously at room temperature for predetermined times, specifically 0 s, 10 min, and 4 h, representing medium and long exchange times, respectively [32]. Then, the exchange reaction was quenched by mixing samples with an equal volume of prechilled quench buffer containing 0.8% (*m*/*v*) formic acid and 0.8 M of guanidine hydrochloride in water, to lower the pH to 2.5, on ice for 1 min. A pH meter was used to determine the pH of the system, with no isotope corrections applied [20].

### 2.6. System Design and Circulation Experiments

**Figure 1:**
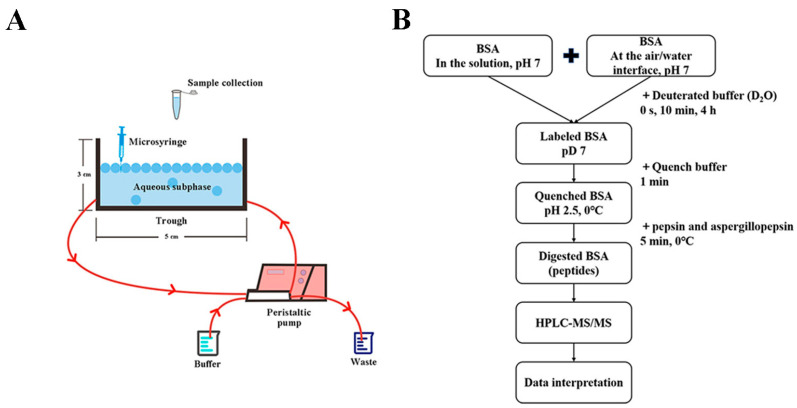
(**A**) Diagram of the device designed for hydrogen–deuterium exchange (HDX) reactions at the air/water interface (the front view); (**B**) schematic representation of the workflow for hydrogen–deuterium exchange mass spectrometry (HDX-MS) of bovine serum albumin (BSA) in the solution and at the air/water interface.

To perform the hydrogen–deuterium exchange experiments at the air/water interface, a self-assembly device was utilized. Several ports were machined into opposite sides of a trough (acrylic), and additional Teflon tubing was connected to a peristaltic pump to efficiently and quickly exchange the aqueous subphase (the buffer under the adsorbed protein layers at the air/water interface) with the D_2_O. Figure 1A displays a schematic drawing of the experimental setup. To measure the minimum amount of subphase buffer needed for full subphase exchange, circulation experiments were conducted. Absorbance measurements of the tartrazine dye-containing buffer at 425 nm were made, both before and after circulation [33]. The efficiency of the subphase exchange was calculated by Equation (4)

(4) $$Exchange\;ratio=\frac{A_{1}}{A_{0}}$$

where A_1_ is the absorbance of the subphase buffer after circulation, and A_0_ is the absorbance of the subphase buffer before circulation.

### 2.7. Hydrogen–Deuterium Exchange at the Air/Water Interface

Hydrogen–deuterium exchange experiments at the air/water interface were carried out utilizing the system described above. An amount of 330 μL of the BSA solution was spread onto the surface of 3 mL of aqueous subphase (PBS, 10 mM, pH 7) in the trough. A microsyringe was used to carry out the procedure of depositing the protein layer at the air/water interface. The protein layer was considered to reach dynamic equilibrium after 10 min [34]. Following that, a peristaltic pump was used to circulate 48 mL of subphase buffer and 12 mL of D_2_O through the trough, which took 5 min and did not disturb the adsorbed proteins at the air/water interface. The adsorbed proteins were labeled for predetermined times—0 s, 10 min, and 4 h—identical to the solution hydrogen–deuterium exchange experiments. Immediately after the labeling period, 300 μL of the adsorbed proteins and subphase buffer (final concentration, 5.78 mg/L) were sucked into a sample tube containing 300 μL of precooling quench buffer.

### 2.8. Digestion, Desalting, and Storage of Samples

The following steps were the same for the quenched samples in the solution and at the air/water interface (Figure 1B). Pepsin and aspergillopepsin (final concentrations 0.020% and 0.023%, *m*/*v*, respectively, dissolved in water) were added, and the samples were digested on ice for 5 min. The digested samples were desalted using Waters Sep-Pak Vac 1cc (50 mg) C18 Cartridges. Finally, the desalted peptides were dried with a CentriVap vacuum concentrator (Labconco, Kansas City, MO, USA) and kept at −80 °C for the following separation and mass analysis.

### 2.9. HPLC-Tandem MS (MS/MS) Analysis, and Data Processing

A homemade 30 cm long pulled-tip analytical column (75 μm ID packed with ReproSil-Pur C18-AQ 1.9 μm resin, Dr. Maisch GmbH) was used to analyze the peptide powder dissolved in 0.1% formic acid. The column set at 55 °C was connected to an Easy-nLC 1200 nano HPLC (Thermo Scientific, Waltham, MA, USA) for mass spectrometry analysis. For peptide separation, the following mobile phase and elution gradient were used: 0.1% formic acid in water as buffer A, and 0.1% formic acid in 80% acetonitrile as buffer B; 0–1 min, 5–10% B; 1–96 min, 10–40% B; 96–104 min, 40–60% B, 104–105 min, 60–100% B, and 105–120 min, 100% B. The flow rate used was 300 nL/min.

Using a Q Exactive Orbitrap mass spectrometer (Thermo Scientific), a data-dependent MS/MS analysis was carried out. A distal 2.5-kV spray voltage was used to electrospray the eluted peptides from the LC column straight into the mass spectrometer. A cycle of one full scan of the MS spectrum (*m*/*z* 300–1800) was obtained. Then, the top 20 MS/MS events were created on the first to the twentieth most intense ions chosen from the full MS spectrum at a 30% normalized collision energy. With an automated gain control (AGC) target of 3 × 10^6^, the full scan resolution was set at 70,000. With an isolation window of 1.8 *m*/*z* and an AGC target of 1 × 10^5^, the MS/MS scan resolution was set at 17,500. For both the MS and MS/MS scans, there was one microscan, and the maximum ion injection times were 50 and 100 ms, respectively. The parameters for the dynamic exclusion were as follows: exclusion time, 30 s; exclude isotopes, on; and charge exclusion, 1 and >8. The Xcalibur data system (Thermo Scientific) managed the LC solvent gradients and MS scan operations. Using the HDExaminer software (Version 3.3, Sierra Analytics), the deuterium incorporation (# Deut, Da) and deuteration percentage (% Deut, %) of each tested peptide at various exchange time points were computed, both of which represented the deuteration level. The deuterium incorporation was the number of deuterons corresponding to the difference between the deuterated and undeuterated centroids, while the deuteration percentage was the ratio of the deuterium incorporation to the theoretical maximum deuteration (Max D). The deuterium incorporation (# Deut, Da) and deuteration percentage (% Deut, %) for each peptide were calculated by Equations (5) and (6), respectively.

(5) $$\#\;Deut=\left(D_{centroid}-UD_{centroid}\right)\times z$$

(6) $$\%\;Deut=\frac{\#\;Deut}{Max\;D}$$

where # Deut represents the deuterium incorporation (Da), D_centroid_ represents the deuterated centroids, UD_centroid_ represents the undeuterated centroids, z is the constant coefficient, % Deut is the deuteration percentage (%), and Max D represents the theoretical maximum deuteration (Da) for each peptide.

The relative deuterium incorporation (r # Deut, Da) of each tested peptide was calculated by subtracting the deuterium incorporation of the peptide in the solution from the deuterium incorporation of the peptide at the air/water interface, while the relative deuteration percentage (r % Deut, %) was calculated by subtracting the deuteration percentage of the peptide in the solution from the deuteration percentage of the peptide at the air/water interface, as indicated by Equations (7) and (8)

(7) $$r\;\#\;Deut=\#\;Deut_{at\;the\;air-water\;interface}-\#\;Deut_{in\;the\;solution}$$

(8) $$r\;\%\;Deut=\%\;Deut_{at\;the\;air-water\;interface}-\%\;Deut_{in\;the\;solution}$$

where r # Deut is the relative deuterium incorporation, and r % Deut is the relative deuteration percentage.

The resulting numbers provided by HDExaminer were then imported into PyMOL (The PyMOL Molecular Graphics System, Version 2.3, Schrodinger, LLC) to map the regions of increased or decreased exchange of BSA.

### 2.10. Statistical Analysis

The statistical analysis was performed with a variance (ANOVA) using the SPSS 26.0 statistical analysis program. The differences were defined through the means of trials by a significant difference test (*p* < 0.05). All the measurements were replicated three times unless stated otherwise.

## 3. Results and Discussion

### 3.1. Optimization of Protein Concentration Used for HDX-MS at the Air/Water Interface and Interfacial Rheology Analysis Based on Foaming Properties

**Figure 2:**
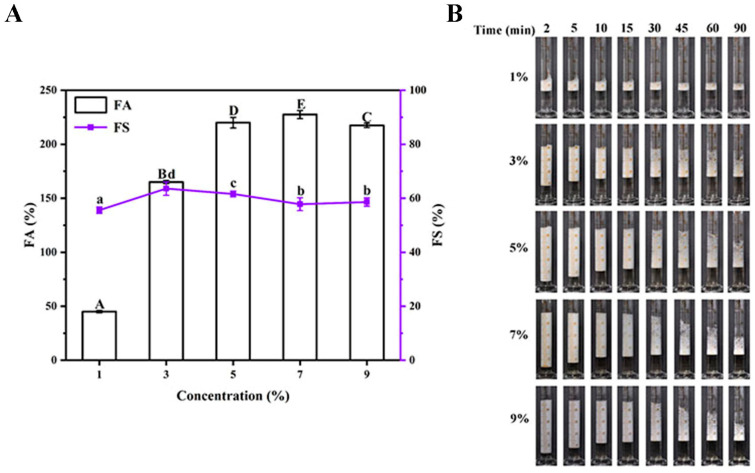
(**A**) Foam ability (FA) and foam stability (FS) of bovine serum albumin (BSA) at different concentrations (1%, 3%, 5%, 7%, and 9%, *m/v*); (**B**) time evolution of the foam volume for different samples. The different uppercase letters represent significant differences (*p* < 0.05) in FA among samples, and the different lower-case letters indicate significant differences (*p* < 0.05) in FS among samples.

The foaming characteristics and foam volume of the BSA under the different concentrations (1, 3, 5, 7, and 9%, *m*/*v*) are presented in Figure 2. Based on Figure 2A, the FA was constant at BSA concentrations from 5% (*m*/*v*) onwards. The FS was not affected by the concentration, which was likely due to the high protein concentration used. With the increase in the BSA concentration, more BSA molecules were adsorbed to the air/water interface, resulting in the ascent of the FA [35,36]. Nevertheless, with the continuous elevation of the protein concentration, the adsorption equilibrium of the BSA at the air/water interface was reached, leading to a constant FA [37,38]. In Figure 2B, the results demonstrate that the foam volume for all the samples steadily reduced from 2 to 90 min. The foaming properties of the BSA were often correlated to the interfacial adsorption and interfacial structures. Considering the results of the FA and FS conjunctively, we decided to use the concentration of 5% (*m*/*v*) in the following interfacial rheology analysis and interfacial HDX-MS experiments.

### 3.2. Determination of the Volume of D_2_O Utilized for HDX-MS at the Air/Water Interface by Circulation Experiments

**Figure 3:**
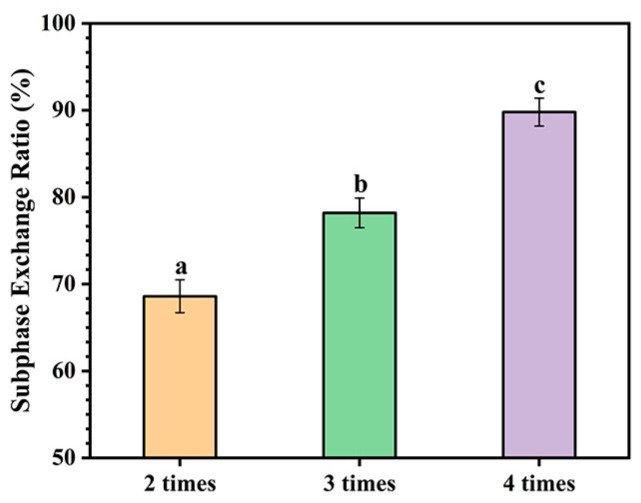
The ratio of subphase exchange with 6 mL (2 times the volume of the subphase buffer in the trough), 9 mL (3 times the volume of the subphase buffer in the trough), and 12 mL (4 times the volume of the subphase buffer in the trough) of fresh buffer. Data denote the mean ± SD of three independent experiments. Significant difference at the *p* < 0.05 level is expressed by different characters at the top of the columns and points.

After the device was assembled, subphase exchange (circulation) experiments were carried out [33]. As shown in Figure 3, the subphase exchange with buffer volumes of 6, 9, and 12 mL (2, 3, and 4 times the volume of the subphase buffer in the trough) resulted in 68.6%, 78.2%, and 89.8% exchange ratios of the subphase, respectively. Although any concentration of D_2_O could be used for HDX experiments, higher concentrations are often used to obtain a higher deuteration level [39]. The 89.8% subphase exchange ratio could meet the requirement of D_2_O concentration (typically 80–90%, *v*/*v*) in the labeling experiments [20]. Moreover, to minimize the structural interference of the protein in the solution on the protein at the interface, 48 mL of fresh buffer was exchanged before the D_2_O to remove the protein in the solution.

### 3.3. Structural Changes of Adsorbed BSA at the Air/Water Interface Analyzed by HDX-MS and Interfacial Rheology

To investigate conformational changes of proteins at the air/water interface, the method of HDX-MS for interfacial proteins was developed. Setting BSA as the model protein, its deuterium incorporation (Da) at the air/water interface and in the solution was compared. Due to its accessibility, BSA has been one of the most studied proteins for decades, contributing to a detailed characterization. With three homologous domains, the three-dimensional structure of BSA is available. In the neutral environment, the percentage of α-helices in BSA is 48% [40]. As a protein, BSA also has ideal functional properties, such as foaming and emulsifying, leading to a wide range of applications in the food field. The available structural information and good features at interfaces for BSA have made it an ideal model of a structured globular protein. Thus, in this study, we adopted BSA as the model protein to analyze protein structural alterations upon adsorption to the air/water interface by HDX-MS.

**Figure 4:**
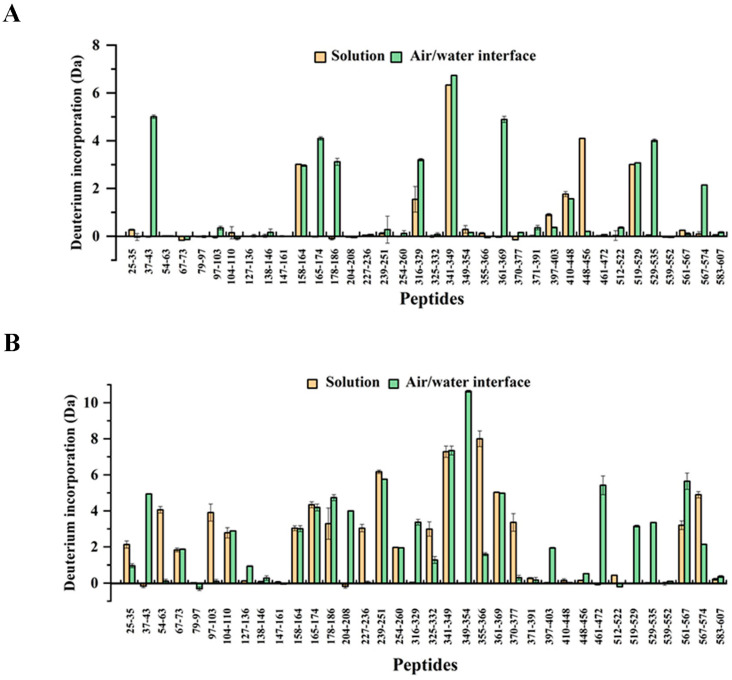
The deuterium incorporation of detected peptides at the (**A**) 10 min labeling time and (**B**) 4 h labeling time, referenced against 0 s. The deuterium incorporation was calculated by the HDExaminer software. Peptides were labeled with the corresponding amino acid numbers.

Our results from the HDX-MS analysis show that, in total, 36 digested peptides of the BSA were detected, leading to a 67.1% sequence coverage (Figure 4). In Figure 4A, the results of the 10 min labeling time showed that the degree of deuterium incorporation for most peptides of the BSA was quite low, both in the solution and at the air/water interface. This was because the rates of deuterium incorporation depended on the stability of the protein structures [25]. The flexible proteins fundamentally tended to show fast deuterium incorporation rates, contributing to a larger mass shift in a shorter labeling time. However, for the typical globular protein, BSA, which contains 17 disulfide bonds and is relatively stable, there were extensive regions with a low deuterium incorporation rate. When the labeling time increased to 4 h (Figure 4B), the majority of BSA peptides showed increased deuterium incorporation. The deuterium incorporation of peptides 54–63, 97–103, 165–174, 227–236, 229–251, 355–366, 361–369, 370–377, 561–567, and 567–574 in the solution labeling at 4 h showed a significant increase compared to the labeling at 10 min. The results indicated that these peptides were located on the surface of the BSA and more exposed to the solvent (D_2_O), which facilitated the adsorption of these peptides to the air/water interface, leading to a certain orientation of BSA at the air/water interface [6]. The peptides 54–63, 165–174, and 355–366 all contained random coil structures, proving the reliability of the HDX-MS results [25]. However, the deuterium incorporation of peptides 448–456 and 519–529 in the solution labeling at 4 h showed a large decrease compared to the labeling at 10 min, as a result of steric solvent shielding (caused by the random protein–protein interaction) and conformational changes [41,42]. As shown in Figure 4, there were significant differences for some peptides in deuterium incorporation between the protein at the air/water interface and in the solution for the interface effect, such as peptides 37–43, 54–63, 97–103, 165–174, 178–186, 204–208, 227–236, 316–329, 349–354, 355–366, 361–369, 370–377, 461–472, 519–529, 529–535, 561–567, and 567–574.

**Figure 5:**
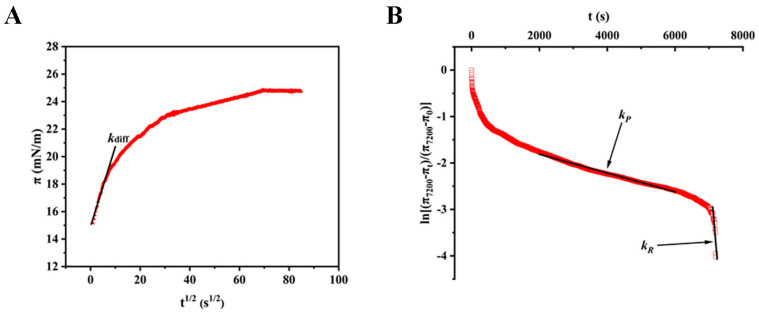
The interfacial rheology of BSA at the air/water interface under the optimal concentration (5%, *m*/*v*). (**A**) Curve of interfacial pressure (π) against the square root of time (t^1/2^). (**B**) A typical plot of ln[(π_7200_ − π_t_)/(π_7200_ − π_0_)] versus time (t).

Meanwhile, the interfacial rheology of BSA was analyzed. After adsorbing to the interface, proteins can greatly lower the interfacial tension to stabilize foam systems. At pH 7.0, the BSA (5%, *m*/*v*) reduced the interfacial tension from 71.85 mNm^−1^ to 45.13 mNm^−1^ after two hours of adsorption. When the adsorption time reached 4000 s, the interfacial tension of the BSA was relatively stable, indicating that this stage was close to the dynamic adsorption equilibrium. The results in Figure 5A show the curve of interfacial pressure (π) versus the square root of time (t^1/2^) for the BSA protein at 5% (*m*/*v*). Within 100 s, the diffusion-controlled fast adsorption process took place. To determine the diffusion rate (k_diff_), the slope of the linear plot of the curve between 0 and 100 s was calculated [0.58 (0.98) mNm^−1^ s^−0.5^]. The high molecular weight of BSA (66 k Da) explains why the value was smaller than that of other proteins [40]. In Figure 5B, the typical plot of ln[(π_7200_ − π_t_)/(π_7200_ − π_0_)] with the time of the BSA at 5% (*m*/*v*) is displayed. The penetration rate (k_P_) of the protein is represented by the slope of the first linear area (2000–6000 s), while its rearrangement rate (k_R_) is shown by the slope of the second linear region (6000–7200 s). The second linear area demonstrates that the BSA completed the structural uncoiling and rearrangement within 2 h. The values of k_P_ and k_R_ were 5.84 × 10^−4^ (0.98) s^−1^ and 18 × 10^−4^ (0.96) s^−1^, respectively, smaller than other flexible proteins, indicating that relatively less conformational rearrangement of the BSA occurred at the air/water interface [43,44].

As analysis of the interfacial rheology results of the BSA testifies, the HDX experiment results of the BSA labeling at 10 min revealed structural changes during the adsorption. Meanwhile, the HDX experiment results of the BSA labeling at 4h reflect the structural state after the uncoiling and rearrangement process.

**Figure 6:**
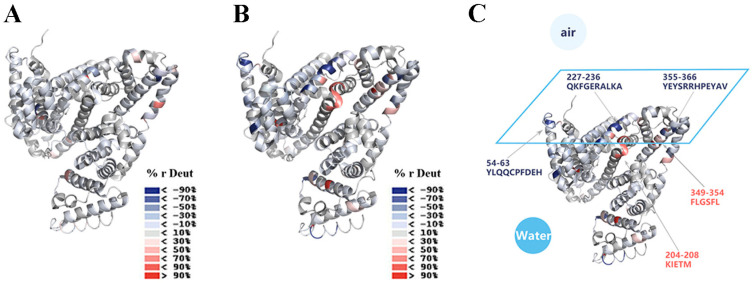
The relative deuteration percentage after (**A**) 10 min labeling and (**B**) 4 h labeling were mapped onto the 3D structures of BSA (PDB: 4F5S), respectively (positive values in red, negative values in blue, as indicated; % r Deut, relative deuteration percentage); (**C**) the plane, on which BSA was supposed to interact with the air/water interface after 4 h labeling, is drawn and the representative peptides are pointed out.

The results of HDX-MS of the BSA in the solution showed that peptide 54–63 exchanged a few deuterium at 10 min labeling, but its deuterium incorporation showed an increase of >4.0 Da at 4 h labeling (Figure 4). However, the same peptide, 54–63, at the air/water interface showed low deuterium incorporation labeling at both 10 min and 4 h, indicating that the air/water interface offered protection from deuterium exchange. Similar results were obtained from the analysis of peptides 227–236 and 355–366. Compared to the BSA in the solution labeling at 4 h, peptide 227–236 at the air/water interface showed a reduction >3.5 Da, and peptide 355–366 demonstrated a decrease >6 Da in the deuterium incorporation (Figure 4B). Figure 6A,B displays the 3D structures of BSA mapped by the relative deuteration percentage labeling at 10 min and 4 h, respectively. From the spatial positions of peptides 54–63, 227–236, and 355–366 in Figure 6C, it was observed that they were on a plane, where the protein interacted with the air/water interface. Thus, we deduced that peptides 54–63, 227–236, and 355–366 were involved in the adsorption of the BSA to the interface, resulting in the certain orientation of the BSA at the air/water interface after 4 h labeling [45]. Our results are in agreement with the current theories: when proteins (especially structured globular proteins) adsorb to the air/water interface, most of the protein molecules remain in the aqueous phase, and only a small part mosaic on the air/water interface [46,47].

Previous research has shown the role of hydrophobic interaction. In that theory, the hydrophobic part of proteins extends to the air phase in the interfacial adsorption [6]. Hydrophobic amino acids (non-polar amino acids, containing Ala, Leu, Ile, Val, Pro, Trp, Phe, and Met) were favorable for protein adsorption to the interface. Moreover, Li et al. [45] examined cryo-electron microscopy information from frozen proteins using various detergents (anionic, cationic, nonionic, and zwitterionic detergents) and found that the electrostatic interaction between proteins and air/water interface carrying negative charges also had an influence on the protein adsorption to the interface. The peptides possessing positively charged amino acid residues (Lys, Arg, and His) were more likely to adsorb to the interface, leading to a preferred orientation of the protein at the interface. Therefore, peptides composed of hydrophobic and positively charged amino acid residues are likely to be involved in the protein adsorption to the air/water interface. These interactions are supposed to affect the orientation of adsorbed proteins on air/water interfaces.

**Figure 7:**
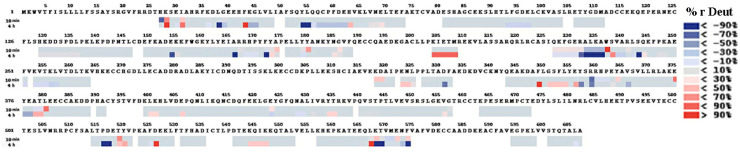
Difference heatmap comparing the deuteration percentages for residues of BSA at the air/water interface versus the deuteration percentages for residues of BSA in the solution. The deuteration percentage in the solution was subtracted from the deuteration percentage at the air/water interface, and the values were colored (positive values in red and negative values in blue, as indicated; % r Deut, relative deuteration percentage).

To determine the residues involved in the interactions between the protein residues and the air/water interface, the relative deuteration percentage of each residue was calculated by subtracting the deuteration percentage of the BSA in the solution from that at the air/water interface. We assumed that the obvious decrease in the residue’s relative deuteration percentage (<−90%) was caused by the interaction between the protein residues and the air/water interface. In our experiment, peptides 54–63, 227–236, and 355–366 showed significant reductions in their relative deuteration percentage. The results in Figure 7 display the difference heatmap denoted by the relative deuteration percentage after labeling at 10 min and 4 h. In peptide 54–63, one positively charged amino acid residue, H63, was located at the end of the peptide. The relative deuteration percentage of L55 was <−90%, which was considered to be involved in the process of adsorption to the air/water interface. For peptide 227–236, the hydrophobic amino acid residues (F229, A233, L234, and A236), and the positively charged amino acid residues (K228, R232, and K235) accounted for 70%. Moreover, the relative deuteration percentages of A233, L234, K235, and A236 were <−90% and the relative deuteration percentage of R232 was <−70%. Peptide 355–366 had three hydrophobic amino acid residues (P362, A365, and V366) and three positively charged amino acid residues (R359, R360, and H361), playing a key role in the adsorption process. Furthermore, the relative deuteration percentage of R359 was <−70%, and the relative deuteration percentage of V366 was <−50%. Thus, residues L55, H63, R232, A233, L234, K235, A236, R359, and V366 from peptides 54–63, 227–236, and 355–366 were considered as the main sites interacting with the air/water interface. The hydrophobic amino acid residues and positively charged amino acid residues accounted for 78% among the amino acid residues, whose relative deuteration percentages were <−70% from peptides 54–63, 227–236, and 355–366, which was consistent with the theory that the protein interacts with the negatively charged air/water interface via hydrophobic and electrostatic interactions.

When the fragments of BSA adsorbed to the air/water interface, the conformation and solvent accessibility of the surrounding peptides were also affected. In Figure 4B and Figure 6C, peptide 204–208, located in the vicinity of peptide 227–236, displayed an increment of >3 Da in the deuterium incorporation on the interface at 4 h labeling. When peptide 227–236 adsorbed to the interface, the conformation of nearby peptides changed because of the interfacial interaction and the interfacial tension. In addition, next to peptide 355–366, peptide 349–354 showed an increased deuterium incorporation, indicating the improvement of its solvent accessibility.

In previous research, the in situ CD spectroscopy technique was utilized to study the conformation (mainly secondary structure) of BSA in the adsorbed state at the air/water interface [16]. The estimation from the surface CD spectrum revealed 35% β-turn, 65% random coil, and no α-helix. Compared with the secondary structure of BSA in the solution, composed of 60% α-helix and 40% random coil, the BSA lost its helix structure on the interface and turned into a disordered protein. This was consistent with our results of far-ultraviolet CD spectroscopy of BSA in the solution in Table S1. In our research, the detected differences in the deuterium incorporation between the samples from the solution and the air/water interface mostly belong to the peptides located in the α-helix structure. Thus, we speculated that the helical structures in peptides 54–63, 227–236, 349–354, and 355–366 transformed into random coils in the process of uncoiling and rearrangement.

Although an air/water interface HDX-MS method was developed to study the conformational changes of interfacial proteins in situ in this study, there was still one shortcoming of the method compared to a standard workflow of HDX-MS experiments [20]. The deuteration rates could be affected by many factors, such as temperature and pH [25]. In order to achieve better separation of peptides, the temperature of the HPLC column was set at 55 °C, which may change the deuteration levels of peptides (back exchange). However, the control samples were set up and the deuteration levels in both the control and experimental samples were affected in the same way. The results in this study, which were calculated by subtracting the deuteration levels of the experimental samples from those of the control samples, have eliminated the effect of back exchange. To improve the sensitivity of the method and increase the intensity of the results further, the temperature of the HPLC column will be set at 0 °C in the following experiments.

## 4. Conclusions

In our project, we successfully established a process of HDX-MS analysis for food systems to monitor the conformational changes of a model protein, BSA, at the air/water interface in situ. Peptides with great differences in deuterium incorporation between the samples in the solution and at the air/water interface were analyzed. The results suggested that the peptides 54–63, 227–236, and 355–366 might be involved in the adsorption of BSA to the air/water interface, and their residues L55, H63, R232, A233, L234, K235, A236, R359, and V366 might interact with the air/water interface through hydrophobic and electrostatic interactions. Furthermore, the conformational changes of peptides 54–63, 227–236, and 355–366 resulted in conformational changes of their surrounding peptides 204–208 and 349–354, causing the reduction of helix structures in the uncoiling and rearrangement process of the interfacial adsorbed BSA. This work could provide a new, powerful method for studying the conformational changes of proteins at the air/water interface and help to understand the mechanism of interfacial protein adsorption and rearrangement at the peptide and amino acid residue level.

## Data Availability

The data presented in this study are available on request from the corresponding author. The data are not publicly available due to privacy.

## Supplementary Materials

The following supporting information can be downloaded at: https://www.mdpi.com/article/10.3390/foods12081601/s1. Table S1. Secondary structure of bovine serum albumin in the solution. Data denote the mean ± SD of three independent experiments.
