# Supplementary material for: The Conformational Changes of Bovine Serum Albumin at the Air/Water Interface: HDX-MS and Interfacial Rheology Analysis
Source: Foods. 2023 Apr 10;12(8):1601. doi: 10.3390/foods12081601 (PMC10137346; doi:10.3390/foods12081601)
Supplement: Supplementary file 1 [file foods-12-01601-s001.zip › foods-2243483-supplementary.pdf]

Supplementary Table S1. Secondary structure of bovine serum albumin in the solution. Data denote the mean ± SD of three independent experiments.

Sample	α-Helix (%)	β-Sheet (%)	β-Turn (%)	Random coil (%)
Bovine serum albumin in solution	59.933±0.368	1.300±0.510	0.567±0.195	38.200±0.356
